# Biomarker-Guided Versus Clinically Guided Management Strategies for Heart Failure: A Systematic Review and Meta-Analysis

**DOI:** 10.31083/RCM46184

**Published:** 2026-03-23

**Authors:** Hao Zhou, Ting Liu, Fuxia Lan, Kai Liu, Xin Wei, Ying Xu

**Affiliations:** ^1^Department of Cardiology, West China Hospital, Sichuan University, 610041 Chengdu, Sichuan, China; ^2^West China Hospital School of Nursing, Sichuan University, 610041 Chengdu, Sichuan, China

**Keywords:** heart failure, brain natriuretic peptide, biomarkers, systematic review, meta-analysis

## Abstract

**Background:**

The clinical value of B-type natriuretic peptide (BNP) or N-terminal pro-B-type natriuretic peptide (NT-proBNP)-guided therapy for improving outcomes in patients with heart failure (HF) remains controversial. Thus, this meta-analysis synthesizes the available evidence from randomized controlled trials (RCTs) to determine whether a biomarker-guided strategy reduces all-cause mortality and HF-related hospitalizations compared with clinically guided management.

**Methods:**

This systematic review followed the Preferred Reporting Items for Systematic Reviews and Meta-Analyses (PRISMA) guidelines. We conducted a systematic search of PubMed, Embase, the Cochrane Library, and Web of Science databases from inception to May 2025 for RCTs comparing biomarker-guided versus clinically guided management in patients with HF. Pooled risk ratios (RRs) were calculated using a random-effects model. We performed extensive supplementary analyses, including a subgroup analysis, sensitivity analysis, and trial sequential analysis (TSA).

**Results:**

We included 17 articles (reporting on 17 distinct RCTs) comprising 5069 patients. The primary meta-analysis showed that biomarker-guided therapy was associated with a significant reduction in all-cause mortality (RR 0.84, 95% confidence interval (CI) 0.73–0.96; I^2^ = 12.2%) and HF-related hospitalizations (RR 0.79, 95% CI 0.65–0.96; I^2^ = 53.7%). However, the robustness of these findings was undermined by subsequent analyses. Meanwhile, a sensitivity analysis restricted to studies with a low risk of bias rendered the mortality benefit non-significant (RR 0.90, 95% CI 0.79–1.03). Egger's test indicated potential publication bias (*p *= 0.0285), and TSA suggested the cumulative evidence was insufficient to draw a definitive conclusion.

**Conclusions:**

Although there is a trend toward benefit, the existing evidence for biomarker-guided HF therapy is deemed “very low” quality based on the Grading of Recommendations, Assessment, Development and Evaluation (GRADE) assessment. The results were compromised by methodological deficiencies in primary studies and potential publication bias. Therefore, the evidence is inadequate to support the routine use of this strategy in clinical practice. Further large-scale, high-quality RCTs are warranted.

**The PROSPERO Registration:**

CRD420250652134, https://www.crd.york.ac.uk/PROSPERO/view/CRD420250652134.

## 1. Introduction

Heart failure (HF) represents a growing global health challenge, affecting an estimated 64 million individuals and imposing a substantial public health and economic burden [1, 2]. Pathophysiologically, HF is defined by congestion or fluid overload, which are the primary drivers of symptom aggravation, organ dysfunction, and recurrent hospitalizations [3, 4]. Despite notable advancements in guideline-directed medical therapy (GDMT), including angiotensin receptor-neprilysin inhibitors (ARNIs) and sodium-glucose cotransporter 2 (SGLT2) inhibitors, hospitalizations for HF remain prevalent, highlighting an ongoing necessity for improved management strategies [5, 6, 7]. Up to fifty percent of patients experience readmission within six months, often as a result of inadequately managed congestion [8].

Traditional fluid management relies on clinical assessment, such as monitoring symptoms and physical signs. These signs are sometimes subjective and not very sensitive, and they usually show up late in the process of hemodynamic deterioration [9, 10]. This can delay required treatment modifications, while overly aggressive diuretic therapy based on these indications may induce adverse outcomes like renal damage and electrolyte abnormalities [11].

B-type natriuretic peptide (BNP) and its N-terminal pro-B-type natriuretic peptide (NT-proBNP) are released from the ventricles in response to increased wall stress, serving as objective and dynamic markers of hemodynamic congestion [12]. Theoretically, titrating HF therapies based on natriuretic peptide levels could enable a more proactive and precise management approach, potentially improving clinical outcomes [13]. However, after more than two decades of investigation, the clinical utility of this strategy remains highly contested. While some trials, like the recent STRONG-HF study, demonstrated that an intensive, NT-proBNP-informed strategy improved outcomes post-discharge for acute HF [14], other large, well-designed trials, most notably GUIDE-IT, found no benefit compared to standard care in high-risk heart failure with reduced ejection fraction (HFrEF) patients [15]. This conflict is further complicated by trials such as TIME-CHF and BATTLESCARRED, which suggested potential age-dependent effects [16, 17].

This evidentiary dissonance has resulted in cautious recommendations from major clinical practice guidelines. Both the 2022 American Heart Association/American College of Cardiology/Heart Failure Society of America (AHA/ACC/HFSA) and 2023 European Society of Cardiology (ESC) guidelines strongly endorse natriuretic peptides for diagnosis and prognostication but decline to issue a Class I recommendation for their use in therapeutic guidance, citing insufficient and conflicting evidence [6, 7]. This creates a critical evidence-practice gap: while biomarker-guided therapy is theoretically attractive for precise management, its inconsistent performance in large RCTs has prevented its clinical adoption. Previous meta-analyses have also yielded inconsistent conclusions, often limited by the inclusion of older, smaller studies [18, 19]. Therefore, this study aims to conduct an updated systematic review and meta-analysis of all eligible randomized controlled trials (RCTs) to clarify whether a biomarker-guided strategy reduces all-cause mortality and HF-related hospitalizations compared to clinically guided management, and to rigorously assess the quality and robustness of the current evidence base.

## 2. Materials and Methods

This systematic review and meta-analysis were conducted and reported following the Preferred Reporting Items for Systematic Reviews and Meta-Analyses (PRISMA) 2020 statement [20]. The study protocol was prospectively registered with the PROSPERO international register of systematic reviews (CRD420250652134).

### 2.1 Literature Search Strategy and Study Selection

We conducted a systematic electronic literature search of PubMed, Embase, the Cochrane Central Register of Controlled Trials (CENTRAL), and Web of Science from their inception to May 2025. The search strategy combined Medical Subject Headings (MeSH) and free-text terms related to “Heart Failure”, “Natriuretic Peptides”, and “Guided Therapy”. The literature screening process was conducted independently by two reviewers. Initially, titles and abstracts were screened, followed by a full-text review of potentially eligible articles to determine final inclusion. Discrepancies were resolved through consensus or by consulting a third reviewer. The full search strategy for all databases is provided in **Supplementary Material 1**.

### 2.2 Inclusion and Exclusion Criteria

Studies were included if they met the following criteria: (1) Study design: Parallel-group RCTs. (2) Participants: Adult patients (age ≥18 years) with a clinical diagnosis of HF. (3) Intervention: Biomarker-guided treatment (BNP or NT-proBNP). (4) Control: Clinically guided standard care. (5) Outcomes: Reported data on all-cause mortality or HF-related hospitalization. We excluded non-randomized studies, reviews, case reports, and conference abstracts without sufficient data.

### 2.3 Data Extraction and Quality Assessment

Two researchers separately extracted data utilizing a standardized form. The extracted data comprised study parameters (author, year, sample size), patient demographics (age, sex, HF type, left ventricular ejection fraction (LVEF)), intervention specifics (biomarker target), follow-up length, and outcome metrics (event counts for each group). The Cochrane Risk of Bias tool 2.0 (RoB 2) (The Cochrane Collaboration, London, UK) was used to rate the overall risk of each RCT as “low risk”, “some concerns”, or “high risk”.

### 2.4 Data Analysis

We performed statistical analyses using R software (version 4.2.1, The R Foundation for Statistical Computing, Vienna, Austria). We calculated pooled risk ratios (RRs) and 95% confidence intervals (CIs) for dichotomous outcomes using a Mantel-Haenszel random-effects model. We quantified heterogeneity via the I^2^ statistic, with I^2^ >50% being considered indicative of significant heterogeneity. We conducted a pre-specified subgroup analysis based on the clinical setting (chronic vs. acute HF) and a sensitivity analysis restricted to studies with a low risk of bias. Publication bias was evaluated using funnel plots and Egger’s test (*p* < 0.1 was considered significant). Trial sequential analysis (TSA) was performed to assess the certainty of the cumulative evidence. Finally, the Grading of Recommendations, Assessment, Development and Evaluation (GRADE) framework was used to assess the overall quality of evidence.

## 3. Results

### 3.1 Literature Search and Study Characteristics

**Fig. 1.**
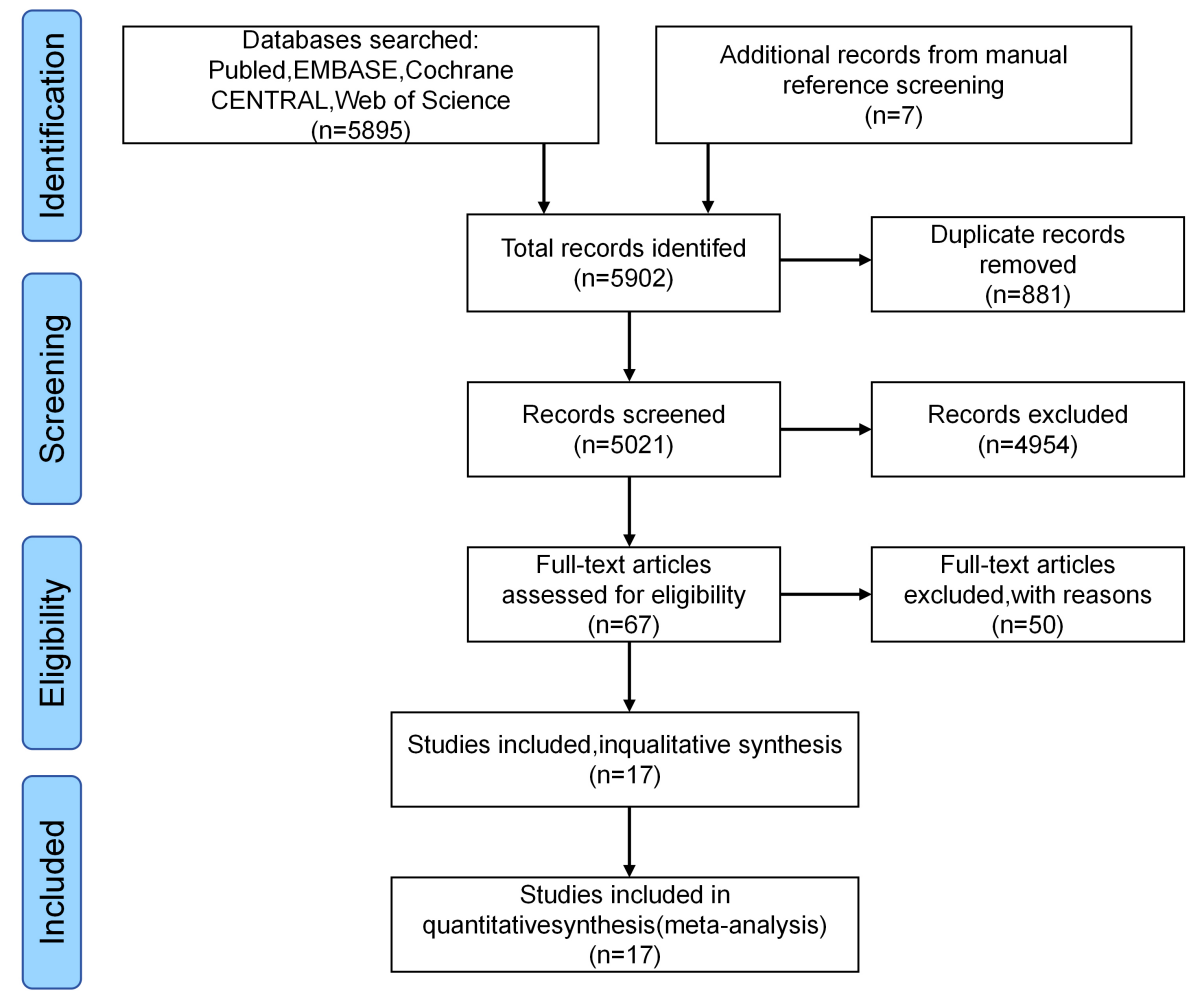
**PRISMA 2020 flow diagram for study selection**. PRISMA, Preferred Reporting Items for Systematic Reviews and Meta-Analyses.

The literature search identified 5895 records. Following multi-stage screening, 17 articles reporting on 17 unique RCTs were included in the final analysis. Cross-verification confirmed no patient overlap. The entire literature screening process is depicted in Fig. 1.

This meta-analysis included 5069 patients (2528 in the biomarker-guided group; 2541 in the clinically guided group). Most trials (n = 14) enrolled patients with chronic HF, while three focused on acute decompensated HF. The majority of trials targeted heart failure with reduced ejection fraction (HFrEF; LVEF <40%) [13, 15], with three studies [17, 21, 22] including mixed LVEF populations or not restricting enrollment by LVEF. No trial exclusively studied HFpEF (LVEF ≥50%), and HFpEF data were sparsely reported across studies. A phenotype-specific subgroup analysis was unfeasible due to limited and inconsistent HFpEF data, limiting generalizability of our findings to this growing patient population. The included studies were published between 2000 and 2023, predominantly conducted in Europe and North America, with follow-up durations ranging from 2 to 18 months. The characteristics of the included studies are detailed in Table 1 (Ref. [13, 14, 15, 16, 17, 21, 22, 23, 24, 25, 26, 27, 28, 29, 30, 31, 32]).

**Table 1.** **Baseline characteristics of included studies**.

Study (authors, year) [Ref]	Trial name/registry ID	N (intervention/control)	Population type	Mean age (years)	LVEF (%)	Follow-up (months)	Biomarker target
Troughton *et al*. (2000) [13]	-	33/36	Chronic HFrEF	69	27	6	NT-proBNP target: decrease
Jourdain *et al*. (2007) [23]	STARS-BNP	110/110	Chronic HFrEF	74	30	15	BNP target: <100 pg/mL
Pfisterer *et al*. (2009) [16]	TIME-CHF	251/248	Chronic HFrEF (≥60 years)	79	30 (Median)	18	NT-proBNP target: <2 × ULN (age-stratified)
Lainchbury *et al*. (2009) [17]	-	66/68	Chronic HFrEF	72	28	10	NT-proBNP target: decrease
Eurlings *et al*. (2010) [24]	PRIMA	151/159	Chronic HF	74	35	12	NT-proBNP target: individual
Januzzi *et al*. (2011) [26]	PROTECT	74/77	Chronic HFrEF	58	25	12	NT-proBNP target: <1000 pg/mL
Karlström *et al*. (2011) [27]	-	66/61	Chronic HFrEF (≥70 years)	81	30	12	BNP target: decrease
Felker *et al*. (2017) [15]	GUIDE-IT	446/448	High-risk HFrEF	62	26	15 (Median)	NT-proBNP target: <1000 pg/mL
Stienen *et al*. (2018) [30]	PRIMA II	204/202	Acute Decompensated HF	76	34 (Median)	6	NT-proBNP target: >30% decrease%
Adamo *et al*. (2023) [14]	STRONG-HF	542/536	Acute HF	64	28 (Median)	6	High-intensity care with NT-proBNP monitoring
Berger *et al*. (2010) [21]	-	40/44	Chronic HF	72	34	9	NT-proBNP-guided
Bajraktari *et al*. (2018) [31]	-	60/60	Outpatient HF	63	34	12	Echo + BNP-guided
Mekontso Dessap *et al*. (2012) [28]	-	151/153	ICU mechanical ventilation with cardiac dysfunction	68	NR	2	NP-driven fluid management
Anguita *et al*. (2010) [22]	-	64/65	Chronic HF	70	36	12	BNP-guided
Kim and Kim (2012) [29]	-	35/35	Chronic HFrEF	57	26	6	BNP-guided beta-blocker titration
Persson *et al*. (2010) [25]	SIGNAL-HF	185/189	Chronic HF (Primary Care)	75	NR	12	NT-proBNP-guided
Saraya *et al*. (2015) [32]	-	50/50	Chronic HFrEF	56	29	6	BNP-guided

Abbreviations: LVEF, left ventricular ejection fraction; HF, heart failure; HFrEF, heart failure with reduced ejection fraction; ULN, upper limit of normal; mgt., management; BB, beta-blocker; BNP, B-type natriuretic peptide; NT-proBNP, N-terminal pro-B-type natriuretic peptide; ICU, intensive care unit.

### 3.2 Risk of Bias Assessment

**Fig. 2.**
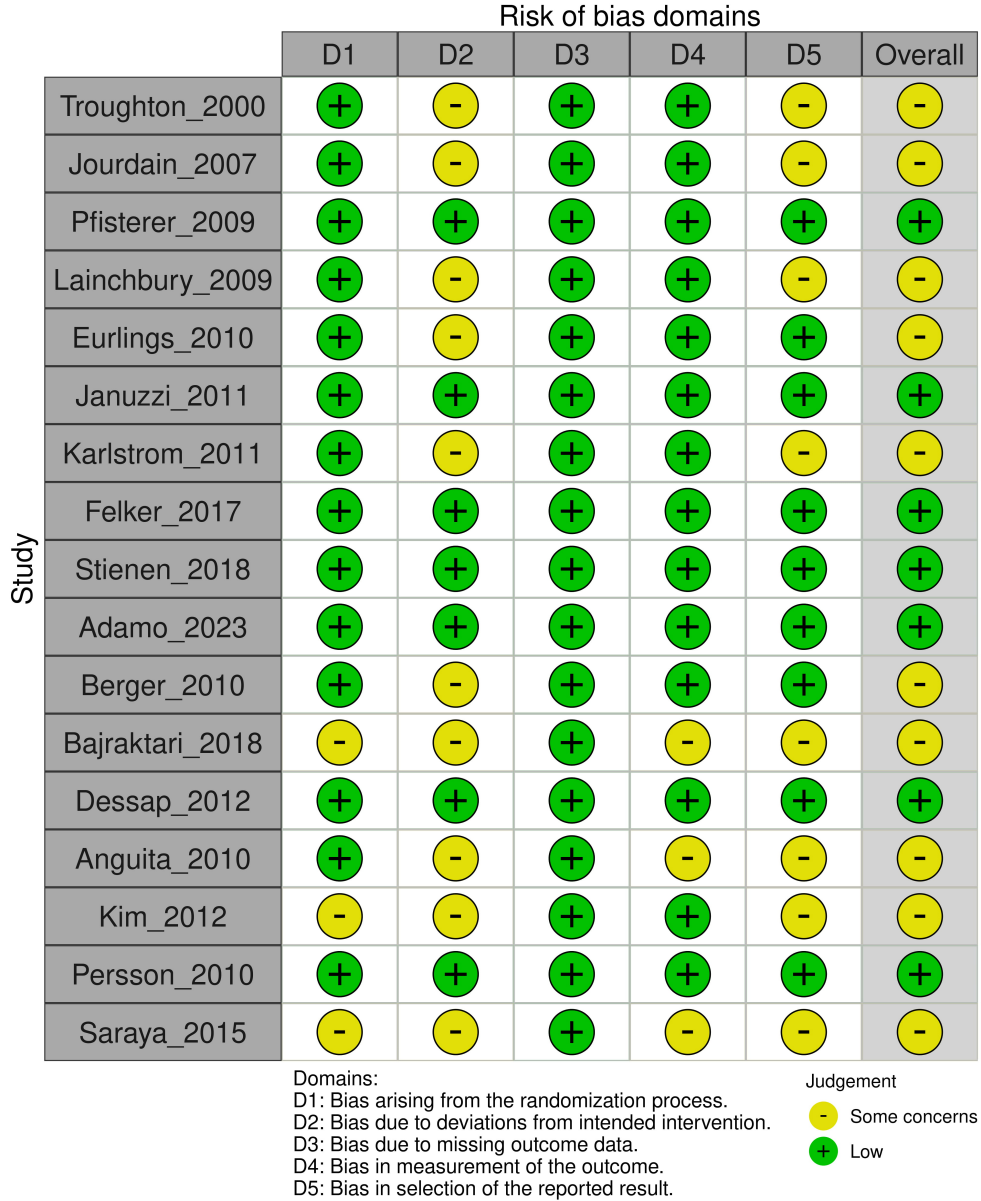
**Risk of bias summary: review authors’ judgements about each risk of bias item for each included study**.

Using the Cochrane RoB 2 tool, we assessed the 17 included studies. Only 7 were rated as having an overall “low risk” of bias. The remaining 10 were rated as having “some concerns”, primarily due to the open-label design of the interventions, which poses a risk of performance bias, and the lack of pre-registered protocols in older studies, which increases the risk of selective reporting bias. The detailed risk of bias assessment is summarized in Fig. 2.

### 3.3 Primary Outcomes

#### 3.3.1 All-Cause Mortality

**Fig. 3.**
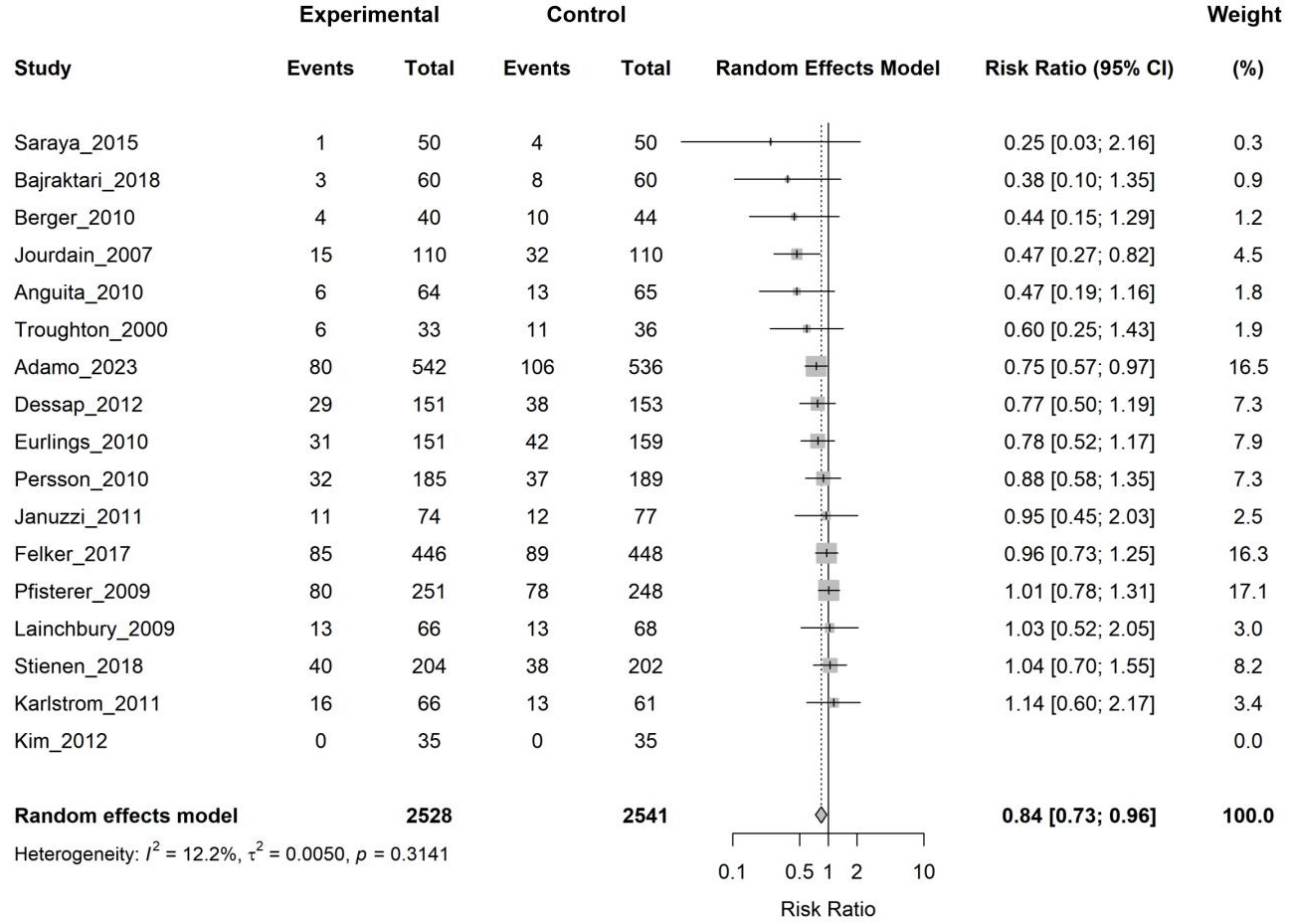
**Forest plot of the effect of biomarker-guided therapy versus clinically guided therapy on all-cause mortality**. CI, confidence interval.

Seventeen studies (5069 patients) reported data on all-cause mortality. The random-effects meta-analysis showed that biomarker-guided therapy was associated with a statistically significant 16% relative risk reduction in all-cause mortality compared to clinical guidance (RR 0.84, 95% CI 0.73–0.96, *p* = 0.015), with low heterogeneity (I^2^ = 12.2%) (Fig. 3).

#### 3.3.2 Heart Failure-Related Hospitalization

**Fig. 4.**
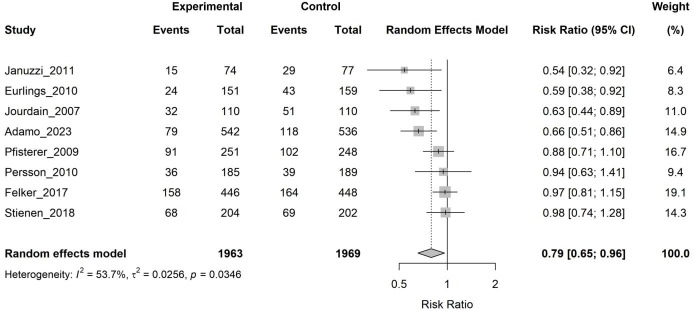
**Forest plot of the effect of biomarker-guided therapy versus clinically guided therapy on heart failure-related hospitalization**.

Eight studies (3932 patients) provided data on HF-related hospitalizations. The pooled analysis demonstrated that the biomarker-guided group had a 21% lower risk of HF hospitalization (RR 0.79, 95% CI 0.65–0.96, *p* = 0.024), though with moderate heterogeneity (I^2^ = 53.7%) (Fig. 4).

### 3.4 Supplementary Analyses

A pre-specified subgroup analysis stratified by clinical setting (chronic vs. acute HF) did not explain the heterogeneity observed for HF-related hospitalization (*p* for subgroup interaction = 0.92).

Critically, a sensitivity analysis restricted to the seven low-risk-of-bias studies showed that the pooled effect for all-cause mortality was no longer statistically significant (RR 0.90, 95% CI 0.79–1.03, *p* = 0.097), underscoring the fragility of the primary finding. Additionally, a leave-one-out sensitivity analysis using the Hartung-Knapp method was performed to challenge the robustness of our findings (**Supplementary Figs. 1,2**). This analysis confirmed our primary results were fragile. For all-cause mortality, omitting the influential Adamo 2023 trial (16.5% weight) caused the result to lose statistical significance (New RR 0.87, 95% CI 0.75–1.004, *p* = 0.056). Similarly, the HF-hospitalization finding also lost significance when several individual studies were omitted (e.g., omitting Jourdain 2007 yielded RR 0.82 [0.66–1.01]). This strongly supports that the ‘naïve’ pooled estimates are not robust and are highly influenced by single studies.

**Fig. 5.**
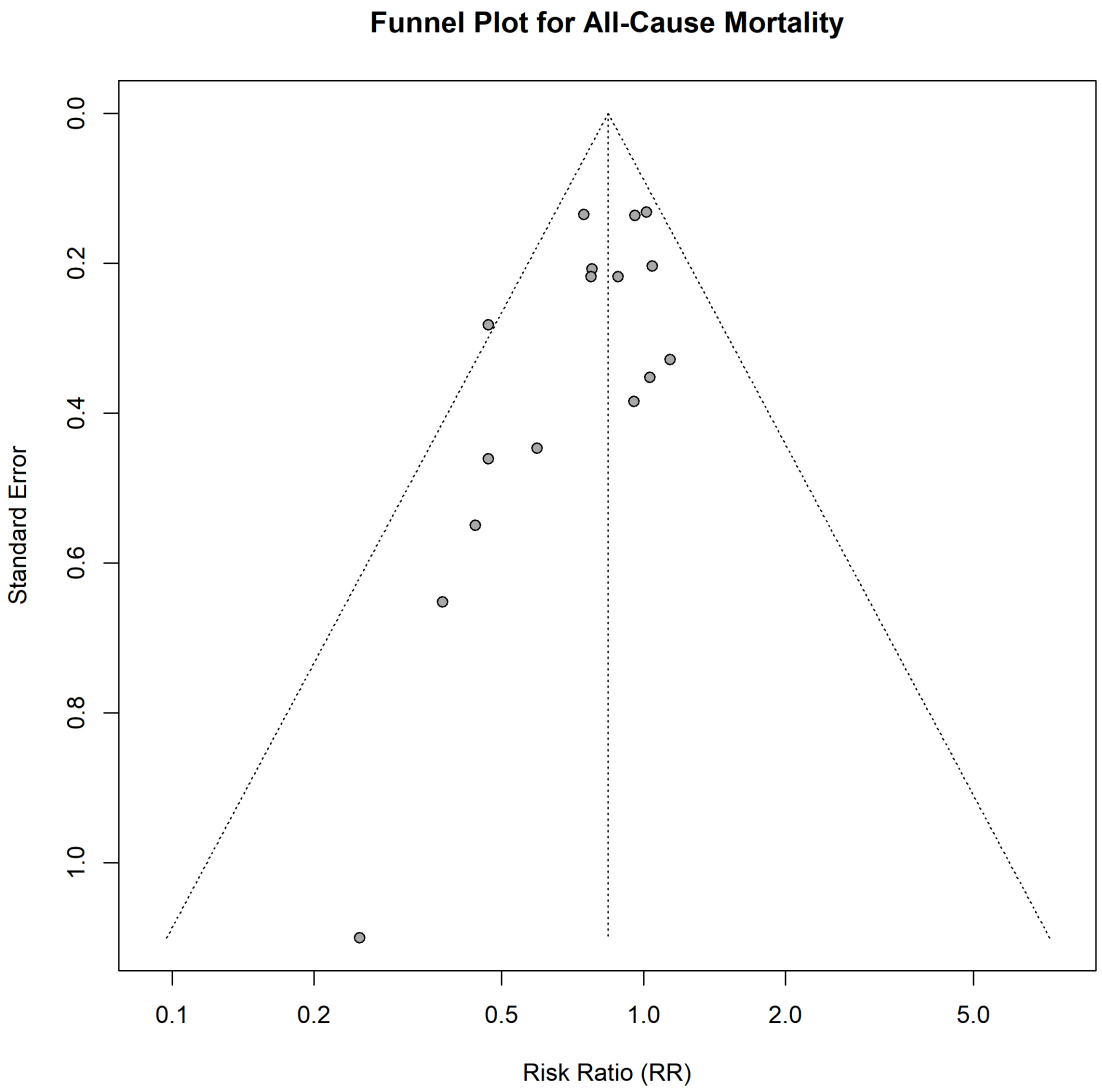
**Funnel plot for the assessment of publication bias for the outcome of all-cause mortality**.

The funnel plot for all-cause mortality was asymmetric (Fig. 5), and Egger’s test confirmed a significant risk of publication bias (*p* = 0.0285).

**Fig. 6.**
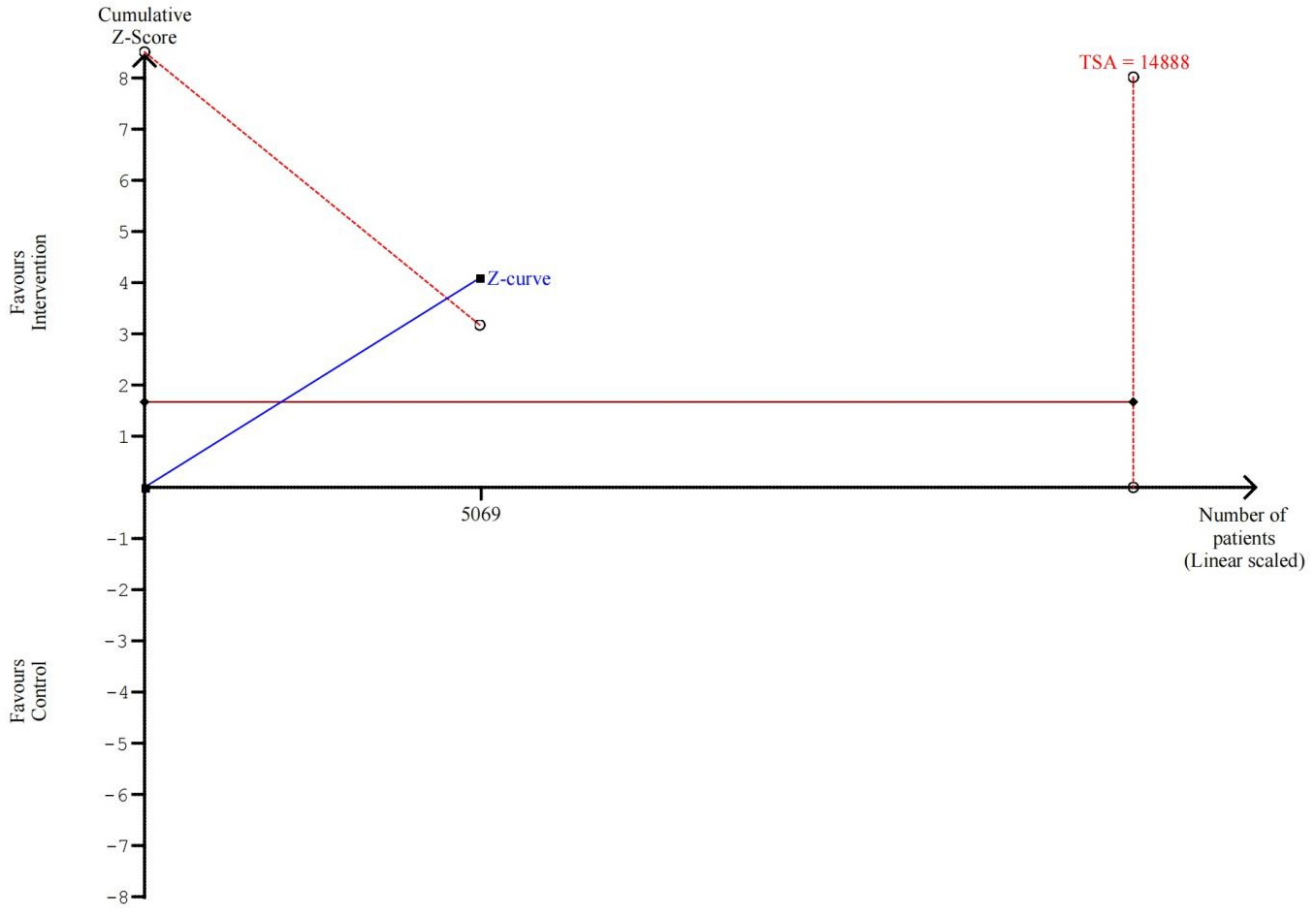
**Trial Sequential Analysis for all-cause mortality**. TSA, trial sequential analysis.

Furthermore, TSA showed that while the cumulative Z-curve crossed the conventional significance boundary, it failed to cross the TSA-defined monitoring boundary for efficacy. The total sample size (5069) was substantially smaller than the required information size (14,888), indicating that the cumulative evidence is insufficient to draw a definitive conclusion (Fig. 6).

## 4. Discussion

The nominal 16% reduction in all-cause mortality (RR 0.84, 95% CI 0.73–0.96) is consistent in direction with previous meta-analyses but must be interpreted with extreme caution [18, 19]. We contend that this ‘naïve’ pooled result is prone to overestimation and does not accurately reflect the intervention’s true clinical benefit. The core issue, illuminated by our sensitivity analysis, is that this mortality benefit disappears entirely when analysis is confined to the most methodologically sound trials (RR 0.90, 95% CI 0.79–1.03). We believe this non-significant finding from the high-quality studies represents the most credible estimate of effect.

This discrepancy strongly suggests that the observed benefit may be an artifact driven by older, smaller, open-label studies which are at high risk of performance bias [33]. In such trials, the “intensified care” effect—whereby patients and clinicians in the intervention arm, aware of the novel strategy, engage more intensively—may contribute more to improved outcomes than the biomarker guidance itself [34, 35]. This concept is supported by the GUIDE-IT trial, which failed to show a benefit, arguably because its control group also received highly structured, intensive clinical follow-up, thereby equalizing the intensity of care between groups [15, 36].

Our conclusion that the primary finding is a false positive (Type I error) is further strengthened by two key analyses. The detection of significant publication bias further weakens the evidence. The tendency for smaller studies with null or negative findings to remain unpublished can create a skewed and overly optimistic representation of an intervention’s efficacy in the published literature [37]. Furthermore, the TSA results provide the most compelling argument against the certainty of the findings, indicating that the cumulative evidence is underpowered and that the statistically significant result from the primary analysis is likely a false positive (Type I error) [38].

The moderate heterogeneity (I^2^ = 53.7%) for the hospitalization outcome likely stems from substantial clinical and methodological diversity across trials [39]. Key sources of heterogeneity include varying natriuretic peptide targets, heterogeneous patient populations (e.g., HFrEF vs. HFpEF, chronic vs. acute), and variable control arm care intensity [15, 40, 41]. The issue of “varying targets” is more problematic than it first appears. Natriuretic peptides are not intrinsically stable metrics. First, they fluctuate significantly within patients and between patients, driven by significant modulation by age, renal function, body mass index (BMI), and comorbidities. Second, different commercial assays produce different readings for the same sample, each with distinct analytical performance and reference ranges. This “noise” from both biological and analytical sources directly fuels what can be termed ‘threshold bias’. A review of Table 1 reveals this lack of consensus: targets ranged from absolute values to relative changes in others. This means the “intervention” was not a uniform strategy across trials. The therapeutic intensity required to meet these disparate goals varied dramatically. We argue this fundamental inconsistency, originating from the biomarker itself and amplified by trial design, is a major, unresolved driver of the heterogeneity we found. As HF is increasingly recognized as a collection of heterogeneous phenotypes, a “one-size-fits-all” biomarker-guided approach may be inherently flawed [42, 43]. Future strategies may need to be tailored to specific patient profiles, potentially integrating multiple biomarkers to capture different pathophysiological domains like inflammation, fibrosis, and renal dysfunction [44, 45].

### 4.1 Clinical Implications and Future Directions

Based on our comprehensive analysis and the resulting “very low” GRADE rating, as detailed in Table 2, the current evidence is insufficient to endorse the routine use of biomarker-guided therapy in clinical practice. The potential benefits do not yet outweigh the uncertainties and the additional resources required [46]. Our findings support the cautious stance of current international guidelines [6, 7].

**Table 2.** **GRADE summary of findings**.

Outcome	Control group risk	Intervention group risk (95% CI)	Relative effect (95% CI)	Absolute effect (per 1000 people)	Quality of evidence (GRADE)
All-cause mortality	214 per 1000	180 per 1000 (156 to 205)	RR 0.84 (0.73–0.96)	34 fewer (9 fewer to 58 fewer)	⊕⊝⊝⊝ Very Low
HF hospitalization	312 per 1000	246 per 1000 (203 to 300)	RR 0.79 (0.65–0.96)	66 fewer (12 fewer to 109 fewer)	⊕⊝⊝⊝ Very Low

Abbreviations: GRADE, Grading of Recommendations, Assessment, Development and Evaluation; RR, risk ratio; CI, confidence interval. GRADE Quality Rating: ⊕⊝⊝⊝ Very Low. Basis for Rating: We initiated the quality rating at ⊕⊕⊕⊕ (High) for RCTs. The quality was downgraded by three levels to ⊕⊝⊝⊝ (Very Low) due to: (1) Serious risk of bias (Downgrade –1) based on the sensitivity analysis and high proportion of ‘some concerns’ studies; (2) Serious publication bias (Downgrade –1) indicated by Egger’s test (*p* = 0.0285); and (3) Serious imprecision (Downgrade –1) confirmed by Trial Sequential Analysis, which showed the required information size was not met.

The path forward requires a new generation of clinical trials that learn from the shortcomings of the past [47]. Future research should focus on: (1) Methodological rigor: To eliminate bias, conduct large-scale RCTs with blinded outcome adjudication [48]. (2) Patient selection: Focus on well-defined, high-risk subgroups (rather than broad HF populations) most likely to benefit, such as those with persistent congestion despite initial therapy [19, 49]. (3) Standardized protocols: Developing and validating clear, actionable, and standardized treatment algorithms linked to specific biomarker changes to ensure interventions are consistent and reproducible [50]. (4) Integration with modern therapies: Evaluating biomarker guidance in the context of contemporary GDMT, including SGLT2 inhibitors, which themselves profoundly impact natriuretic peptide levels and volume status [51, 52].

### 4.2 Strengths and Limitations

This review’s strengths include a comprehensive search method and the use of advanced statistical techniques, such as TSA and the GRADE framework, to critically appraise the evidence and estimate the certainty of the overall conclusions. However, the quality of the original research included in the analysis limits the conclusions. The identified risks of bias, severe publication bias, and statistical imprecision are major limitations.

## 5. Conclusions

In summary, the prospective benefit of biomarker-guided therapy in HF is suggested by a pooled analysis of existing RCTs; however, this conclusion is based on very low-quality evidence and lacks robustness. Prevalent methodological flaws, statistical imprecision, and a high risk of publication bias erode confidence in the effect estimate. This combination of factors leads to our conclusion that the current evidence is insufficient to support the routine implementation of this strategy. There is a clear and urgent need for large-scale, methodologically rigorous RCTs to definitively define the role, if any, of biomarker-guided therapy in contemporary HF management.

## Availability of Data and Materials

All data generated or analyzed during this study are included in this published article.

## Supplementary Material

Supplementary material associated with this article can be found, in the online version, at https://doi.org/10.31083/RCM46184.
