# Supplementary material for: Biomarker-Guided Versus Clinically Guided Management Strategies for Heart Failure: A Systematic Review and Meta-Analysis
Source: Rev Cardiovasc Med. 2026 Mar 23;27(3):46184. doi: 10.31083/RCM46184 (PMC13036551; doi:10.31083/RCM46184)
Supplement: Supplementary file 1 [file 2153-8174-27-3-46184-s1.zip › Supplementary Figures.docx]

**
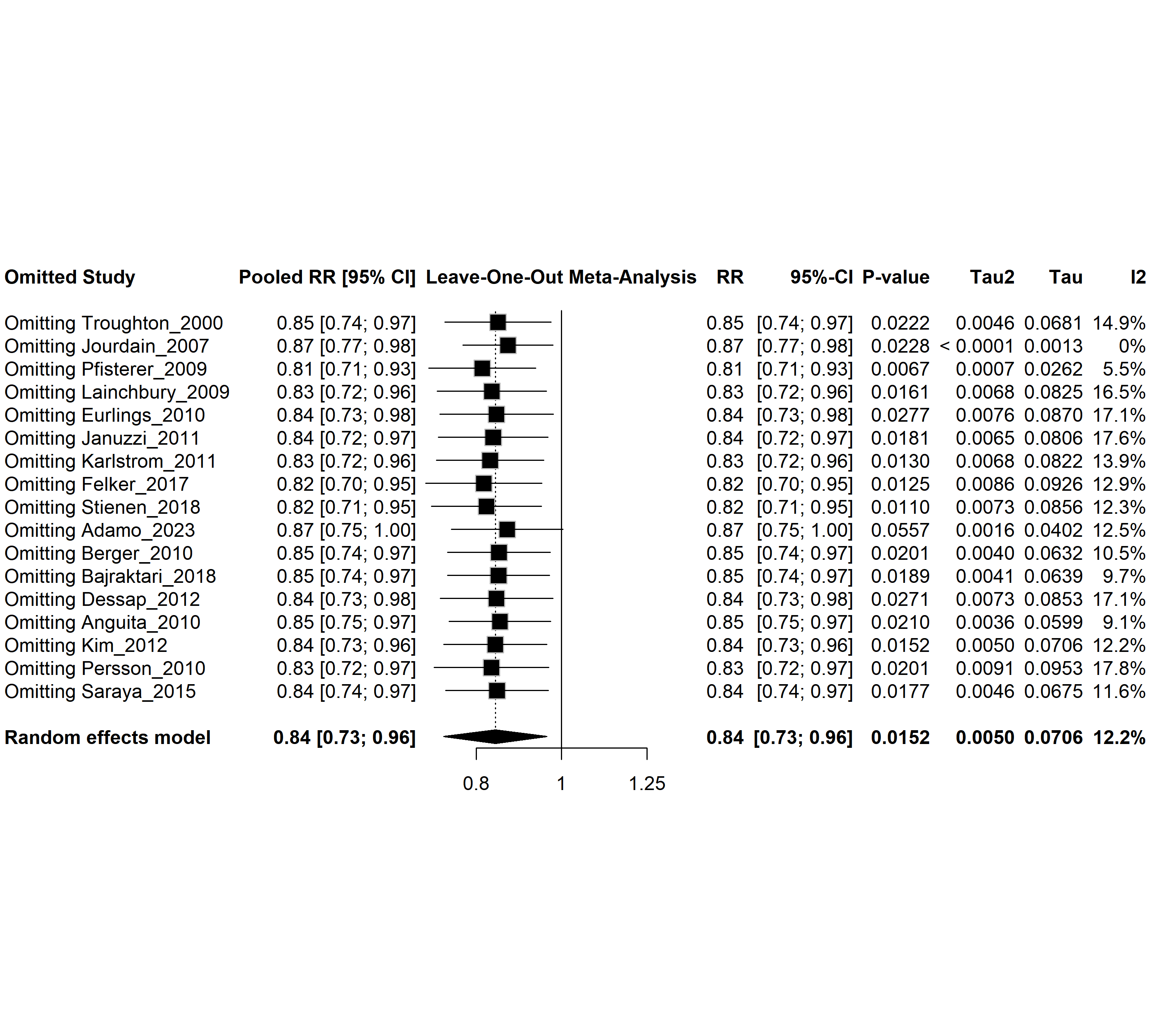
Supplementary Fig. 1. Leave-one-out sensitivity analysis for all-cause mortality.** Each row (black squares) represents the pooled Risk Ratio (RR) and 95% Confidence Interval (CI) calculated by omitting the single study listed on the left. The black diamond indicates the original overall pooled RR from the random-effects model (**RR 0.84, 95% CI: 0.73–0.96**), including all 17 studies. The vertical line at 1.0 indicates no effect.

**
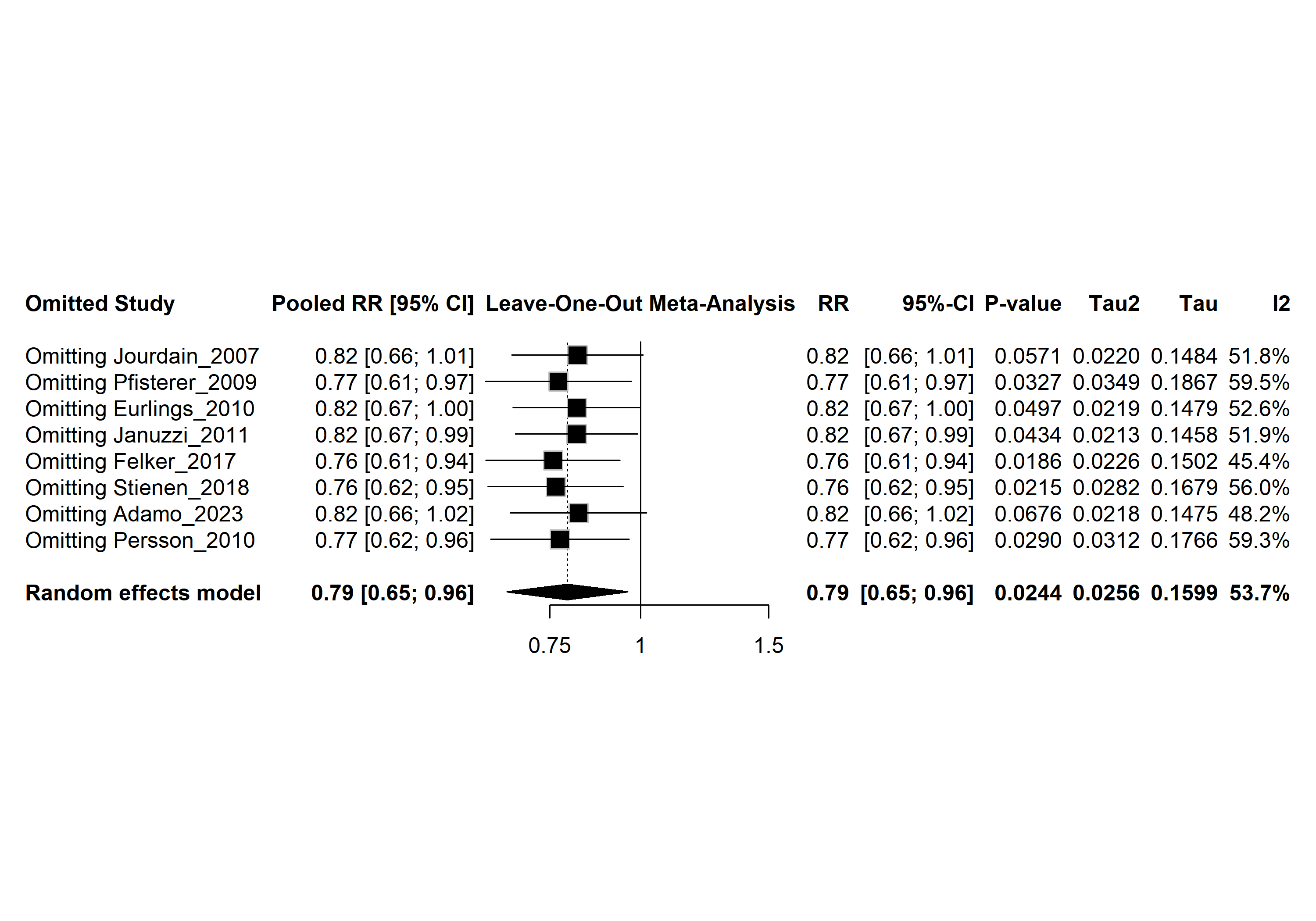
**

**Supplementary Fig. 2. Leave-one-out sensitivity analysis for HF-related hospitalization.** Each row (black squares) represents the pooled Risk Ratio (RR) and 95% Confidence Interval (CI) calculated by omitting the single study listed on the left. The black diamond indicates the original overall pooled RR from the random-effects model (**RR 0.79, 95% CI: 0.65–0.96**), including all 8 studies. The analysis demonstrates that the omission of any single study did not significantly alter the pooled estimate, confirming the robustness of the finding.
