# Supplementary material for: Biomarker-Guided Versus Clinically Guided Management Strategies for Heart Failure: A Systematic Review and Meta-Analysis
Source: Rev Cardiovasc Med. 2026 Mar 23;27(3):46184. doi: 10.31083/RCM46184 (PMC13036551; doi:10.31083/RCM46184)
Supplement: Supplementary file 1 [file 2153-8174-27-3-46184-s1.zip › Supplementary Material 1 Full Search Strategy.docx]

### Supplementary Material 1: Full Search Strategy

Database: PubMed

Search executed on: May 30, 2025

Filters: Randomized Controlled Trial

#1 (("Heart Failure"[Mesh] OR "Cardiac Failure"[tiab] OR "Heart Failure"[tiab] OR "Heart Decompensation"[tiab] OR "Ventricular Dysfunction"[tiab]))
#2 (("Natriuretic Peptide, Brain"[Mesh] OR "BNP"[tiab] OR "B-Type Natriuretic Peptide"[tiab] OR "NT-proBNP"[tiab] OR "pro-BNP"[tiab] OR "proBNP"[tiab] OR "Natriuretic Peptide"[tiab]))
#3 (("Fluid Therapy"[Mesh] OR "Diuretics"[Mesh] OR "guided"[tiab] OR "directed"[tiab] OR "tailored"[tiab] OR "monitored"[tiab] OR "adjusted"[tiab] OR "Fluid Management"[tiab] OR "Fluid Removal"[tiab] OR "Decongestion"[tiab] OR "Diuretic Therapy"[tiab]))
#4 #1 AND #2 AND #3
#5 "Randomized Controlled Trial"[Publication Type] OR "Controlled Clinical Trial"[Publication Type] OR "randomized"[Title/Abstract] OR "placebo"[Title/Abstract] OR "trial"[Title]
#6 #4 AND #5

Database: Embase

Search executed on: May 30, 2025

Filters: Randomized Controlled Trial

#1 ('heart failure'/exp OR 'cardiac failure':ti,ab OR 'heart failure':ti,ab OR 'heart decompensation':ti,ab OR 'ventricular dysfunction':ti,ab) AND ('brain natriuretic peptide'/exp OR 'bnp':ti,ab OR 'b type natriuretic peptide':ti,ab OR 'nt-probnp':ti,ab OR 'pro-bnp':ti,ab OR 'probnp':ti,ab OR 'natriuretic peptide':ti,ab) AND ('fluid therapy'/exp OR 'diuretic agent'/exp OR 'guided':ti,ab OR 'directed':ti,ab OR 'tailored':ti,ab OR 'monitored':ti,ab OR 'adjusted':ti,ab OR 'fluid management':ti,ab OR 'fluid removal':ti,ab OR 'decongestion':ti,ab OR 'diuretic therapy':ti,ab) [cite: 4]
#2 ('brain natriuretic peptide' OR bnp OR 'b type natriuretic peptide' OR 'nt-probnp' OR 'pro-bnp' OR probnp OR 'natriuretic peptide') NEAR/5 ('fluid therapy' OR 'diuretic agent' OR guided OR directed OR tailored OR monitored OR adjusted OR 'fluid management' OR 'fluid removal' OR decongestion OR 'diuretic therapy') [cite: 5]
#3 #1 AND #2 [cite: 6]

Database: Web of Science

Search executed on: May 30, 2025

Filters: Randomized Controlled Trial

#1 TS=("Heart Failure" OR "Cardiac Failure" OR "Heart Decompensation" OR "Ventricular Dysfunction")
#2 TS=(("BNP" OR "B-Type Natriuretic Peptide" OR "NT-proBNP" OR "pro-BNP" OR "proBNP" OR "Natriuretic Peptide") NEAR/5 ("guided" OR "directed" OR "tailored" OR "management" OR "therapy" OR "strategy" OR "treatment" OR "decongestion" OR "fluid removal" OR "diuretic therapy"))
#3 #1 AND #2

Database: Cochrane Library (CENTRAL)

Search executed on: May 30, 2025

Filters: Randomized Controlled Trial

#1 ([mh "Heart Failure"] OR "Cardiac Failure":ti,ab OR "Heart Failure":ti,ab OR "Heart Decompensation":ti,ab OR "Ventricular Dysfunction":ti,ab) [cite: 10]
#2 ([mh "Natriuretic Peptide, Brain"] OR [mh "Natriuretic Peptides, B-Type"] OR "BNP":ti,ab OR "B-Type Natriuretic Peptide":ti,ab OR "NT-proBNP":ti,ab OR "pro-BNP":ti,ab OR "proBNP":ti,ab OR "Natriuretic Peptide":ti,ab) [cite: 11]
#3 ([mh "Fluid Therapy"] OR [mh "Diuretics"] OR "guided":ti,ab OR "directed":ti,ab OR "tailored":ti,ab OR "monitored":ti,ab OR "adjusted":ti,ab OR "Fluid Management":ti,ab OR "Fluid Removal":ti,ab OR "Decongestion":ti,ab OR "Diuretic Therapy":ti,ab) [cite: 12]
#4 #1 AND #2 AND #3 [cite: 13]
