# Supplementary figures and images for: Biomarker-Guided Versus Clinically Guided Management Strategies for Heart Failure: A Systematic Review and Meta-Analysis
Source: Rev Cardiovasc Med. 2026 Mar 23;27(3):46184. doi: 10.31083/RCM46184 (PMC13036551; doi:10.31083/RCM46184)

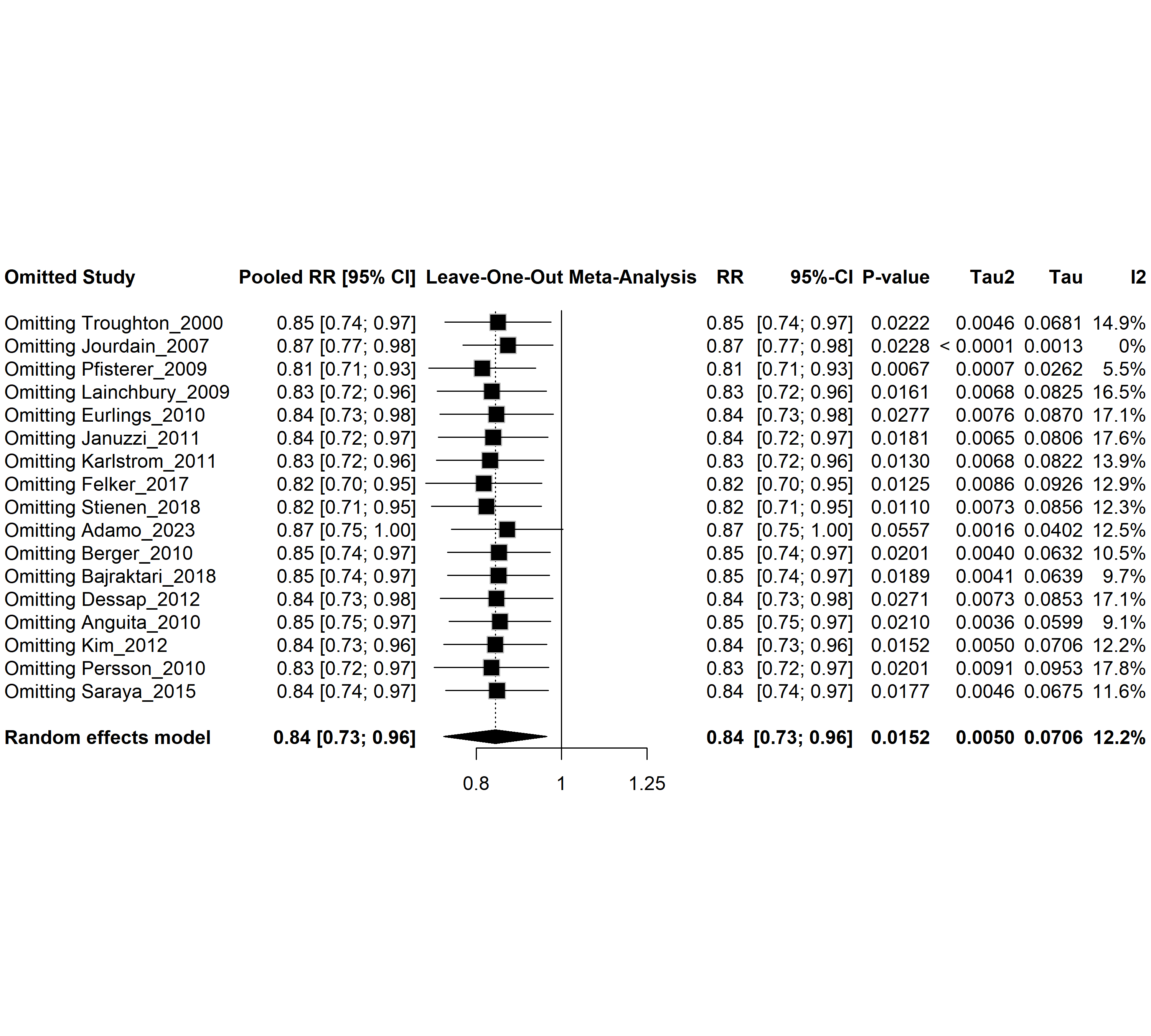

Supplement: Supplementary file 1 [file 2153-8174-27-3-46184-s1.zip › Supplementary Fig. 1_L-O-O_Mortality.png]

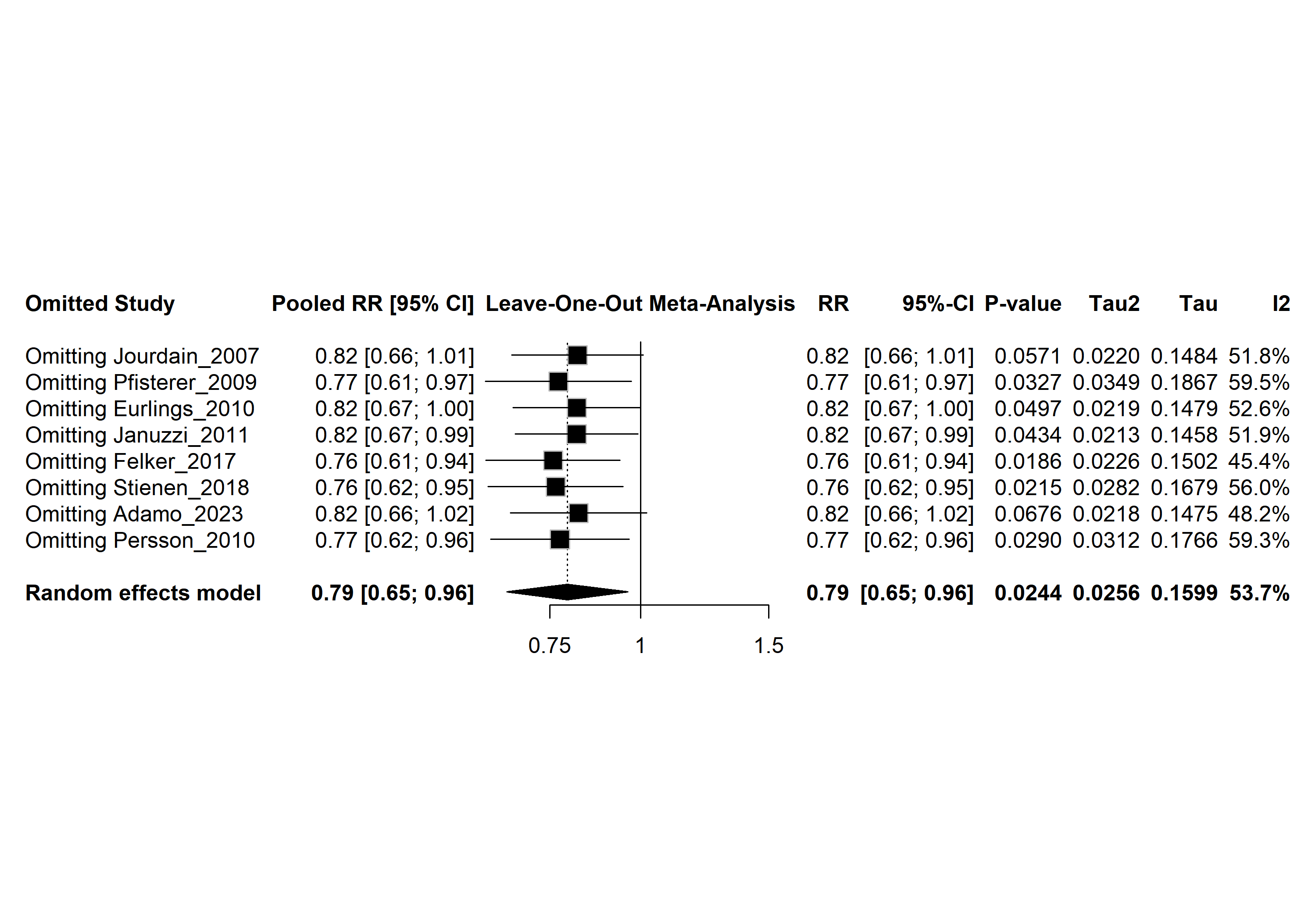

Supplement: Supplementary file 1 [file 2153-8174-27-3-46184-s1.zip › Supplementary Fig. 2_L-O-O_HF_Hospitalization.png]
